# Cardiac embolization of an implanted fiducial marker for hepatic stereotactic body radiotherapy: a case report

**DOI:** 10.1186/1752-1947-3-140

**Published:** 2009-11-20

**Authors:** Hooman Hennessey, David Valenti, Tatiana Cabrera, Valerie Panet-Raymond, David Roberge

**Affiliations:** 1 Department of Oncology, Division of Radiation Oncology, McGill University Health Centre, 1650 Cedar Avenue, Montreal, Quebec, H3G 1A4, Canada; 2 Department of Radiology, Division of Interventional Radiology, McGill University Health Centre, 1650 Cedar Avenue, Montreal, Quebec, H3G 1A4, Canada

## Abstract

**Introduction:**

In liver stereotactic body radiotherapy, reduction of normal tissue irradiation requires daily image guidance. This is typically accomplished by imaging a surrogate to the tumor. The surrogate is often an implanted metal fiducial marker. There are few reports addressing the specific risks of hepatic fiducial marker implantation. These risks are assumed to be similar to percutaneous liver biopsies which are associated with a 1-4% complication rate - almost always pain or bleeding. To the best of our knowledge, we present the first case of such a fiducial marker migrating to the heart.

**Case presentation:**

An 81-year-old Caucasian man (5 years post-gastrectomy for a gastric adenocarcinoma) was referred post-second line palliative chemotherapy for radiotherapy of an isolated liver metastasis. It was decided to proceed with treatment and platinum fiducials were chosen for radiation targeting. Under local anesthesia, three Nester embolization coils (Cook Medical Inc., Bloomington, IN, USA) were implanted under computed tomography guidance. Before the placement of each coil, the location of the tip of the delivery needle was confirmed by computed tomography imaging. During the procedure, the third coil unexpectedly migrated through the hepatic vein to the inferior vena cava and lodged at the junction of the vena cava and the right atrium. The patient remained asymptomatic. He was immediately referred to angiography for extraction of the coil. Using fluoroscopic guidance, an EN Snare Retrieval System (Hatch Medical L.L.C., Snellville, GA, USA) was introduced through a jugular catheter; it successfully grasped the coil and the coil was removed. The patient was kept overnight for observation and no immediate or delayed complications were encountered due to the migration or retrieval of the coil. He subsequently went on to be treated using the remaining fiducials.

**Conclusion:**

Implanted fiducial markers are increasingly used for stereotactic radiotherapy. There is sparse literature on the risks of such procedures. Although uncommon, the risk of migration does exist and therefore physicians (surgeons, oncologists and radiologists) and patients should be aware of this possibility.

## Introduction

Stereotactic body radiation therapy (SBRT) is a technique that utilizes precise targeting of tumor irradiation in order to minimize exposure of adjacent normal tissue [1]. SBRT is used to treat a variety of primary and metastatic tumors of the lung, liver, pancreas, kidney, spine and prostate [2]. In the use of SBRT for liver lesions, tumor-targeting accuracy is crucial given the radiosensitive nature of the liver, frequent proximity of the target to the small bowel as well as significant displacement of the liver with breathing.

In hepatic SBRT, reduction of normal tissue irradiation requires image guidance [1]. This is typically accomplished through daily imaging of a surrogate to the tumor. The surrogate can be the whole liver, the diaphragm or an implanted marker. The imaging modality can be stereoscopic fluoroscopy, pretreatment megavoltage or kilovoltage imaging, cone-beam computed tomography (CT) or 'CT on rails'. Depending on the combination used, implanted fiducials may or may not play a role. Although non-fiducial-based strategies avoid an invasive procedure, they do not typically permit imaging during treatment or 'tracking' of the tumor. At our center, we have used pretreatment megavoltage images of implanted fiducials for targeting and continuous portal imaging during treatment for quality assurance. While flexible helical gold coils are commonly used [3] as fiducials, our access to such products had been limited by the lack of local regulatory approval. We have found that platinum embolization coils can be a safe and effective alternative for tumor localization in stereotactic liver radiotherapy [4]. These coils are easily inserted percutaneously with a small caliber needle during a well-tolerated outpatient procedure.

Percutaneous fiducial implantation is minimally invasive but does have risks and limitations [5]. The most commonly described complications involve implantation of fiducials for lung SBRT. When pulmonary fiducials are placed percutaneously, pneumothoraces are common, while pulmonary hemorrhage is rare. There are limited data specific to hepatic implantation. In a series of 21 patients implanted with 2 mm gold spheres, one of the fiducials migrated through the inferior vena cava to a small vein at the hip where it remained lodged without apparent clinical consequence [6]. In a series from Stanford University Medical Center, 33 patients underwent CT-guided placement of 0.8 mmx5 mm hepatic fiducials. In these cases, there was no gross marker migration and one patient experienced a small hemorrhagic pleural effusion [7]. Comparison with the liver biopsy literature may provide a benchmark to estimate the risk of hepatic fiducial implantation, as the techniques are similar. In the extensive experience of percutaneous liver biopsies, the reported risk of complications is 1 to 4% [8]. Serious complications are almost always pain or bleeding (or sequelae thereof).

Fiducial migration is a twofold issue, first of tumor mistargeting as a consequence of fiducial migration within the targeted organ and second of potential direct toxicity from the marker exiting the targeted organ.

Migration of fiducials within the targeted organ has been described. Most published work concerns prostate radiotherapy with studies looking at the relative position of three to four prostate markers as a surrogate to migration [9]. In limited experience with hepatic fiducials, intra-organ migration appears minimal -- in the order of 2 to 3 mm [6,9]. However, quantification of movement within the liver is limited by the frequent lack of precise landmarks within this large, deformable organ. To deal with this issue, clinicians have relied on treatment margins, repeated three-dimensional imaging and/or implantation of redundant fiducials.

Migration of seeds placed in the prostate for radiotherapy is common. Most commonly, these prostate seeds do not migrate acutely and are found in the lung on follow-up imaging [10]. They can also migrate in the pelvis or abdomen as well as being expressed through the urethra. These incidents are generally without complication although an acute cardiac event due to migration into a coronary artery has been described [11]. In the chest, benign migration of fiducials can be seen into the airway or pleural space. To the best of our knowledge, we report the second case of extra-hepatic migration of a liver fiducial but the first case where an implanted hepatic fiducial marker for SBRT targeting embolized to the chest.

## Case presentation

An 81-year-old Caucasian man had been diagnosed 5 years earlier with a gastric adenocarcinoma for which he underwent surgery. At the time, adjuvant radiotherapy was considered but was deferred when a liver metastasis was discovered. The patient was treated with palliative chemotherapy. He had an excellent treatment response and was subsequently followed without treatment for 2 years before undergoing radiofrequency ablation for progression of his hepatic lesion. After four cycles of second line palliative chemotherapy, the patient, still in excellent general condition, was referred for SBRT as consolidation treatment of what remained a metabolically active isolated liver metastasis. It was decided to proceed with treatment and platinum fiducials were chosen for SBRT targeting. Under local anesthesia, Nester embolization coils (Cook Medical Inc., Bloomington, IN, USA) were implanted using a 21-gauge needle under CT guidance. Each coil is 14 cm long, with a diameter of 0.889 mm and an approximate configuration of 11.7 loops creating an *in vivo *embolus of 4 mm.

For our patient, three coils were placed by an experienced interventional radiologist: the first on the anterior and medial border of the lesion, the second on the anterior lateral border and the third on the posterior border of the lesion. Before the placement of each coil, the location of the tip of the delivery needle was confirmed by CT imaging. On the immediate post-deployment CT, the third coil was not seen in the liver. A scout CT image localized it in the chest. During the procedure, the coil had unexpectedly migrated through the hepatic vein to the inferior vena cava (IVC) and lodged at the junction of the IVC and the right atrium. The patient was asymptomatic but was immediately referred to angiography for extraction of the coil.

**Figure 1:**
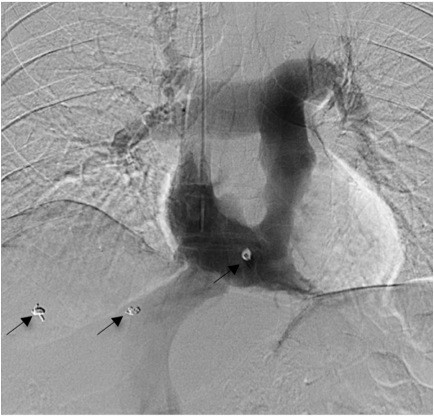
**Digital subtraction angiogram with the catheter trip within the right atrium**. Arrows depict fiducial coil markers.

Using ultrasound guidance and accessing through the right internal jugular vein, a guide wire was inserted over which a straight multi-side hole catheter was inserted and an angiogram was performed with the catheter tip within the right atrium (Figure 1).

**Figure 2:**
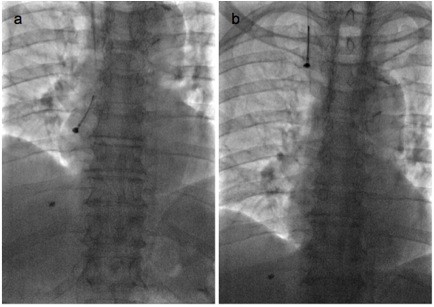
**EN Snare retrieving coil from the right atrium (a) and passing through the superior vena cava, (b) under fluoroscopic guidance**.

Using fluoroscopic guidance, an EN Snare Retrieval System (Hatch Medical L.L.C., Snellville, GA, USA) was used. The tip of the catheter was slightly curved and inserted over the guide wire toward the coil within the right atrium. The EN Snare was introduced through the catheter; it successfully grasped the coil, and the coil was removed through the sheath (Figure 2). The patient was kept overnight for observation and no immediate or delayed complications were encountered due to the migration or retrieval of the coil.

Shortly after, it was possible to proceed with SBRT with daily image guidance based on the previously implanted platinum fiducials. Unfortunately, a short time thereafter, the patient developed intra-hepatic bile duct dilatation. This occurred secondary to documented tumor progression and was unrelated to the fiducial placement. The patient underwent percutaneous biliary drainage and was admitted to hospital for end of life care.

## Discussion

Implanted markers have been shown to improve radiotherapeutic precision and provide a target for tumor tracking. Although SBRT can be performed without fiducials, these are mandatory for specific radiosurgery systems [12]. Previous groups have principally used gold seeds as fiducial markers [6,7]. We have reported our initial experience using platinum embolization coils as markers for hepatic SBRT localization [4]. Platinum has an electron density similar to that of gold and, in our experience, platinum coils are a reasonable alternative to gold seeds or baguettes. Moreover, platinum coil insertion has generally been well tolerated by our patients with post-implantation transient hepatalgia as the only complication in 20 to 25% of patients.

The platinum coils have inherent thrombogenic properties through their embedded synthetic fibers, which may help to minimize the risk of bleeding post-insertion. They are inserted with a small caliber (21G), minimally traumatic needle thus enabling the procedure to be carried out on an outpatient basis. Gold fiducials for megavoltage imaging can require a biopsy caliber needle for insertion. Thinner gold fiducials can be used if kilovoltage imaging is available, although these smaller markers may be more prone to migration. Given the coiled configuration, platinum embolization coils are thin enough to require a small caliber needle for insertion and yet large enough to create a readily visible 4 mm radiodense target. By its nature and spiral configuration, migration of this marker was thought to be unlikely. However, in this case report, we have shown that migration is a possible complication. The third coil implanted in this patient's liver migrated through the hepatic vein to the IVC where it lodged at the junction of the IVC and the right atrium and subsequently had to be removed via catheterization. Fortunately, the marker did not reach the pulmonary vasculature where it may have caused a significant infarct.

For migration to occur, we believe that the needle tip would have to have been inserted into a hepatic vein branch with a diameter of several millimeters. In a vein of this caliber, there is typically blood return when the needle stylet is withdrawn. In our patient, there was no blood return. This leads us to postulate that the needle tip was in parenchyma very close to an appropriately sized vein and either the needle migrated or the act of deploying the marker led to perforation of the vein wall. With deployment, the coil likely ended in an intravascular location and was carried to the IVC by normal venous flow, highlighting the importance of immediate post-deployment imaging.

In our patient, a risk factor for migration was the proximity of the tumor to a major hepatic vein. In such cases, the risk may be mitigated by determining the location of regional veins on pre-implantation contrast-enhanced images and planning a safe approach. We now routinely use intravenous contrast for all marker placements in the superior portion of the liver, especially if in close proximity to a hepatic vein. Placement under ultrasound guidance is an alternative, although we find it convenient to have the possibility, in a single procedure, of acquiring a contrast CT unencumbered by artifact immediately before fiducial placement for use in treatment planning.

In stereotactic radiotherapy of pulmonary tumors, endovascular fiducial placement has been used as a means of reducing the risk of a pneumothorax [13]. In the liver, the additional risks and inconveniences of formal angiography would appear out of proportion to the small risk of migration.

## Conclusion

Interventional radiologists and radiation oncologists often collaborate in the implantation of radio-opaque markers for the targeting of focused radiation. There is sparse literature on the risks of such procedures. Although we have found the use of platinum embolization coils to be a generally safe and effective option for tumor localization in SBRT, complications may occur. To the best of our knowledge, we report the first case of cardiac migration of an implanted hepatic radiotherapy fiducial marker. Although unlikely, the risk of migration does exist and physicians and patients should be aware of this risk.

## Abbreviations

CT: computed tomography; IVC: inferior vena cava; SBRT: stereotactic body radiotherapy.

## Consent

This case report has been prepared in accordance to hospital, provincial and federal guidelines, rules and laws. The patient who is described in this case is deceased. The case itself focuses on an unreported complication of a procedure and not the patient's disease. The patient is not identified in the case report, no identifying information is used and all radiology images have been anonymized. A statement from the department head and the institution director of professional services can be provided approving the use of this patient's medical information for the purpose of the case report.

## Competing interests

The authors declare that they have no competing interests.

## Authors' contributions

DV helped write the manuscript, provided background pertinent to radiologists and contributed to the interpretation and discussion of the case. TC performed the procedure described, provided a description of the events, helped write the manuscript and provided context appropriate for radiologists. VPR reviewed the literature pertinent to radiotherapy fiducials and helped write the background to the manuscript. DR was a major contributor in writing the manuscript in addition to having the supervisory role in this report. HH investigated and compiled the patient's medical chart and was a major contributor in writing the manuscript. All authors read and approved the final manuscript.
